# Liver Fibrosis and IRS1 rs2972146 Are Associated With Weight Loss Response After Metabolic Bariatric Surgery

**DOI:** 10.1111/dom.71345

**Published:** 2026-09-17

**Authors:** Felice Cinque, Marica Meroni, Sara Badiali, Miriam Longo, Erika Paolini, Elisa Galfrascoli, Giovanna Oberti, Giuseppina Pisano, Gabriele Maffi, Matteo Mureddu, Elena Corbetta, Valentina Scimè, Emma Calzavara, Francesca Alletto, Annalisa Cespiati, Luigi Boni, Anna Ludovica Fracanzani, Paola Dongiovanni, Rosa Lombardi

**Affiliations:** ^1^ SC Medicina ad Indirizzo Metabolico Fondazione IRCCS Cà Granda Ospedale Maggiore Policlinico Milan Italy; ^2^ Department of Pathophysiology and Transplantation University of Milan Milan Italy; ^3^ Department of General and Minimally‐Invasive Surgery Fondazione IRCCS Ca' Granda Ospedale Maggiore Policlinico Milan Italy

**Keywords:** genetic susceptibility, insulin resistance, MASH, MASLD, obesity, weight regain

## Abstract

**Background and Aims:**

Weight loss after metabolic bariatric surgery (MBS) is heterogeneous, and the contribution of liver histology and genetic susceptibility to insufficient weight loss (IWL) or weight regain (WR) remains unclear. We assessed the prevalence of IWL and WR after MBS and their association with liver histology and metabolic dysfunction‐associated steatotic liver disease (MASLD)‐related genetic variants.

**Methods:**

We retrospectively included 351 adults undergoing MBS at Policlinico Hospital in Milan, between 2008 and 2023, with intraoperative liver biopsy. IWL was defined at 18 months as < 50% excess weight loss and/or BMI ≥ 35 kg/m^2^, WR as ≥ 10% increase from postoperative weight nadir. PNPLA3, TM6SF2, GCKR, HSD17B13, and IRS1 variants were genotyped.

**Results:**

Out of 351 patients, 109 (31.1%) had IWL and out of 280 patients with available data, 91 (32.5%) developed WR. Hypertension (OR 1.97, 95% CI: 1.06–3.63) and significant liver fibrosis (OR 2.93, 95% CI: 1.07–8.01) were positively independently associated with IWL, whereas the IRS1 rs2972146 variant (OR 0.30, 95% CI: 0.14–0.62), sleeve gastrectomy or gastric bypass (OR 0.20, 95% CI: 0.10–0.39), dietary (OR 0.32, 95% CI: 0.16–0.63) and physical activity adherence (OR 0.38, 95% CI: 0.16–0.89) were inversely associated with IWL. WR was associated with psychiatric comorbidity and inversely associated with preoperative BMI, sleeve gastrectomy/gastric bypass, and dietary adherence.

**Conclusions:**

Weight loss failure affected nearly one third of patients after MBS. Significant liver fibrosis and IRS1 rs2972146 variant may help identify patients at risk of suboptimal postoperative weight loss outcomes.

## 1 Introduction

Obesity is a chronic, relapsing disease affecting more than 1 billion people worldwide [1] and driving metabolic, cardiovascular, and liver‐related morbidity, including metabolic dysfunction‐associated steatotic liver disease (MASLD) and liver fibrosis [1, 2, 3].

Current obesity treatments include lifestyle, pharmacological, endoscopic, and surgical approaches [4, 5]. Metabolic bariatric surgery (MBS) remains the most effective strategy for substantial and sustained weight loss in severe obesity [6, 7, 8], and is recommended for individuals with BMI ≥ 40 kg/m^2^ or BMI ≥ 35 kg/m^2^ with obesity‐related comorbidities [9].

However, 15%–35% of patients experience weight loss failure, including insufficient weight loss (IWL), commonly defined as < 50% excess weight loss and/or body mass index (BMI) ≥ 35 kg/m^2^ at 18 months [10, 11], and weight regain (WR), generally defined as ≥ 10% regain from postoperative nadir [12]. However, definitions have varied considerably across studies. To standardize reporting and avoid stigmatizing terminology, the 2024 International Federation for the Surgery of Obesity and Metabolic Disorders (IFSO) consensus defined a suboptimal initial response as < 20% total weight loss or inadequate improvement in a clinically significant obesity complication [13]. Late postoperative clinical deterioration was defined as recurrent weight gain exceeding 30% of the initial weight lost or worsening of a significant obesity complication after an initially adequate response [13]. These definitions facilitate comparability and clinical interpretation across studies.

Potential determinants of suboptimal outcomes include baseline BMI, metabolic and psychiatric comorbidity, eating behaviour, physical inactivity, follow‐up adherence, procedure‐related factors, and genetic susceptibility, in particular variants involved in obesity susceptibility, adipogenesis, thermogenesis, insulin signalling, and eating behaviour [10, 14, 15, 16].

Despite growing evidence linking preoperative comorbidities to postoperative weight loss failure [17], the role of liver disease and genetic susceptibility remains poorly defined. In particular, whether histological features of MASLD or liver disease–related genetic variants influence postoperative weight loss response remains unclear.

Therefore, this study aimed to assess the prevalence and factors associated with IWL and WR after MBS, with a specific focus on liver‐related factors and genetic susceptibility. We specifically investigated whether histological MASLD severity, including MASH and fibrosis, and selected MASLD‐ and insulin resistance–related genetic variants were associated with suboptimal postoperative weight loss outcomes.

## 2 Patients and Methods

### 2.1 Patients Cohort

We retrospectively analysed 351 consecutive adults who underwent MBS at Fondazione IRCCS Ca′ Granda Ospedale Maggiore Policlinico, Milan, Italy, between January 2008 and December 2023. Eligibility for MBS followed Società Italiana di Chirurgia dell'Obesità e delle Malattie Metaboliche (SICOB) recommendations and regional criteria [9]. In brief, MBS was indicated for patients with BMI ≥ 40 kg/m^2^, irrespective of comorbidities, and for those with BMI 35–39.9 kg/m^2^ and at least one obesity‐related complication. Selected patients with class I obesity (BMI 30–34.9 kg/m^2^) were also considered eligible when at least one major obesity‐related complication was not adequately controlled by medical therapy. Patients were included if they had an intraoperative liver biopsy and 18‐month postoperative anthropometric data sufficient to assess IWL.

### 2.2 Surgical Procedures

Bariatric procedure selection was based on multidisciplinary evaluation following counselling on benefits, risks, complications, and long‐term follow‐up, in accordance with SICOB recommendations [9]. Procedures included sleeve gastrectomy, gastric bypass, adjustable gastric banding, and gastric plication. Sleeve gastrectomy was generally offered to patients without severe gastroesophageal reflux disease or large hiatal hernia and to those requiring future endoscopic gastric surveillance. Gastric bypass was preferred in patients with clinically relevant gastroesophageal reflux disease or T2DM because of its greater effects on reflux and glycemic control. Gastric banding and plication were mainly performed in selected patients during the earlier study years, reflecting historical practice. All procedures were performed by experienced bariatric surgeons using standard techniques.

### 2.3 Clinical and Biochemical Assessment

Baseline demographic, anthropometric, lifestyle, clinical, pharmacological, and biochemical data were collected at the time of preoperative evaluation.

BMI was calculated as weight/height^2^ and patients were categorized as lean (BMI < 25 kg/m^2^), overweight (BMI 25–29.9 kg/m^2^), class I obesity (BMI 30–34.9 kg/m^2^), class II obesity (BMI 35–39.9 kg/m^2^), or class III obesity (BMI ≥ 40 kg/m^2^). T2DM was defined as fasting plasma glucose ≥ 126 mg/dL on at least two determinations, random plasma glucose and/or 2‐h plasma glucose after oral glucose load ≥ 200 mg/dL, glycated haemoglobin ≥ 48 mmol/mol, previous diagnosis, or use of glucose‐lowering medication [18]. Hypertension was defined as in‐office blood pressure ≥ 140/90 mmHg or use of antihypertensive therapy [19]. Dyslipidemia was defined as low‐density lipoprotein cholesterol (LDL‐C) > 100 mg/dL, or lower according to individual Systematic COronary Risk Evaluation 2 cardiovascular risk, triglycerides > 150 mg/dL, high‐density lipoprotein cholesterol (HDL‐C) < 40 mg/dL in men or < 50 mg/dL in women, or use of lipid‐lowering therapy [20]. Homeostatic model assessment insulin resistance index (HOMA‐IR) > 2.5 was diagnostic for insulin resistance [18]. Biochemical parameters included alanine aminotransferase (ALT), aspartate aminotransferase (AST), gamma‐glutamyl transferase (GGT), fasting glucose, glycated haemoglobin, lipid profile, thyroid function.

Lifestyle data included dietary and physical activity adherence. Dietary adherence was assessed according to bariatric dietitian recommendations. Physical activity adherence was defined as 150–300 min/week of moderate aerobic activity, 75–150 min/week of vigorous activity, or resistance training of all major muscle groups at least twice weekly [21]. Smoking, previous intragastric balloon procedures, psychiatric comorbidities—including depressive, anxiety, anxiety–depressive, eating, and personality disorders—psychotropic medication use, and antidiabetic or weight‐management therapies were recorded.

### 2.4 Assessment of Postoperative Weight Loss Outcomes

The primary outcomes were IWL and WR [10, 11]. IWL was assessed in patients with 18‐month postoperative weight data, whereas WR was assessed in those with available postoperative nadir and subsequent follow‐up weight. Accordingly, IWL and WR were analysed as distinct outcomes, reflecting poor early weight loss response and later recurrence of weight after nadir, respectively.

IWL was defined at 18 months according to Reinhold criteria as < 50% EWL and/or postoperative BMI ≥ 35 kg/m^2^. EWL percentage was calculated as: %EWL = [(preoperative weight−postoperative weight)/(preoperative weight−ideal body weight)] × 100, where ideal body weight was defined as the weight corresponding to a BMI of 25 kg/m^2^ [10, 11].

WR was defined as a ≥ 10% increase in body weight from the lowest postoperative weight achieved after surgery [12].

Because standardized longitudinal data on changes in obesity‐related complications were unavailable, the complete clinical definitions proposed by the 2024 IFSO consensus [13] could not be applied retrospectively. We therefore performed sensitivity analyses using their weight‐based components. Weight‐defined suboptimal initial response was defined as < 20% total weight loss at 18 months. Weight‐defined late postoperative clinical deterioration was defined as recurrent weight gain > 30% of the maximum weight initially lost following an adequate initial response and achievement of postoperative nadir. Percentage recurrent weight gain was calculated as follows: [(follow‐up weight−nadir weight)/(preoperative weight−nadir weight)] × 100 [13].

Lifestyle adherence, psychiatric comorbidities, psychotropic medication use, and current treatments were recorded at outcome assessment.

### 2.5 Liver Assessment

Intraoperative liver biopsies were reviewed by one expert pathologist according to the Kleiner classification [22, 23]. The NAFLD Activity Score (NAS; 0–8) combined steatosis, lobular inflammation, and ballooning. MASH was defined as NAS ≥ 5 with ballooning. Fibrosis was staged F0–F4; significant fibrosis was ≥ F2, advanced fibrosis ≥ F3, and fibrotic MASH was MASH with fibrosis > F1 [22, 23].

Serum AST, ALT, and GGT levels were interpreted according to local laboratory cutoffs, with values considered elevated when > 39 U/L for AST, ≥ 41 U/L for ALT, and ≥ 61 U/L in men or > 36 U/L in women for GGT.

### 2.6 Genetic Analysis

Patients were genotyped for rs738409 C>G (patatin‐like phospholipase domain‐containing protein 3 [*PNPLA3*] p.I148M), rs58542926 C>T (transmembrane 6 superfamily member 2 [*TM6SF2*] p.E167K), rs1260326 C>T (glucokinase regulator [*GCKR*] p.P446L), rs72613567:TA (hydroxysteroid 17‐beta dehydrogenase 13 [*HSD17B13*]) and rs2972146 C>T (insulin receptor substrate 1 [IRS1]) variants using duplicate TaqMan assays (QuantStudio 3, ThermoFisher), as described [24]. Genotyping success exceeded 99%. Variants were selected for associations with MASLD, progressive liver disease, or insulin resistance [25, 26, 27, 28, 29], and analysed by risk/protective allele carriage.

### 2.7 Statistical Analysis

Continuous variables were summarized as mean ± SD or median (IQR), and categorical variables as *n* (%). Groups were compared using Mann–Whitney *U*, chi‐square, or Fisher's exact tests. Clinically relevant variables or those associated with outcomes in univariable analyses entered multivariable logistic regression models. Results are reported as ORs with 95% CIs. Two‐sided *p* < 0.05 was significant. Sensitivity analyses were conducted using the weight‐based components of the 2024 IFSO definitions for suboptimal initial response and late postoperative clinical deterioration. To assess potential temporal heterogeneity arising from changes in bariatric practice, analyses were also stratified by surgical era (2008–2016 and 2017–2023). Analyses used IBM SPSS Statistics.

The study was approved by the Ethics Committee of Fondazione IRCCS Ca′ Granda Ospedale Maggiore Policlinico, Milan, Italy (Study ce 401). The research was conducted in accordance with the principles of the Declarations of Helsinki. All participants provided informed consent before enrollment in the research studies conducted at their respective sites.

## 3 Results

### 3.1 Cohort Characteristics

A total of 351 patients with available IWL data after MBS were included (Table 1). The mean age was 43.7 ± 10.5 years, and most patients were female (77.2%). Mean preoperative BMI was 42.9 ± 6.0 kg/m^2^; 33.3% had BMI 35–40 kg/m^2^ and 63.3% BMI ≥ 40 kg/m^2^. The most frequent metabolic comorbidities were insulin resistance (85.2%), dyslipidemia (47.6%), impaired fasting glucose (34.7%), hypertension (34.1%), and T2DM (13.1%). The most common bariatric procedures were gastric banding (45.0%), gastric bypass (40.7%), and sleeve gastrectomy (11.4%). Previous intragastric balloon procedures were recorded in 95 patients (27.1%). Histologically, MASLD was present in 300 patients (85.5%), MASH in 43 (12.2%), and fibrotic MASH in 32 (9.1%). Fibrosis stage ≥ 1 was observed in 139 patients (39.6%), while significant fibrosis stage ≥ 2 was present in 26 patients (7.4%).

**TABLE 1:** Demographic, lifestyle, anthropometric, metabolic, and procedural characteristics according to insufficient weight loss and weight regain status.

Variables	Whole cohort (*n* = 351)	IWL (*n* = 109)	No IWL (*n* = 242)	*p*	WR (*n* = 91)^a^	No WR (*n* = 189)^a^	*p*
Demographics and lifestyle							
Age, years	43.7 ± 10.5	45.3 ± 10.9	43.0 ± 10.2	0.058	42.8 ± 9.8	44.8 ± 10.4	0.127
Female sex, *n* (%)	271 (77.2)	84 (77.1)	187 (77.3)	0.966	74 (81.3)	142 (75.1)	0.248
Smoking, *n* (%)	84 (23.9)	29 (26.6)	55 (22.7)	0.487	25 (27.5)	41 (21.7)	0.305
Adherence to diet at 18 months, *n* (%)	191 (54.4)	45 (41.3)	146 (60.3)	< 0.001	—	—	
Adherence to diet at last follow‐up^b^, *n* (%)	154 (55.0)	—	—		30 (33.0)	124 (65.6)	< 0.001
Adherence to physical activity at 18 months, *n* (%)	102 (29.0)	14 (12.8)	88 (36.4)	< 0.001	—	—	
Adherence to physical activity at last follow‐up^b^, *n* (%)	81 (28.9)	—	—		21 (23.0)	60 (31.7)	0.142
Psychiatric comorbidity, *n* (%)	36 (10.2)	18 (16.5)	18 (7.4)	0.017	16 (17.6)	15 (7.9)	0.012
Anthropometry							
Preoperative BMI, kg/m^2^	42.9 ± 6.0	45.8 ± 7.4	41.6 ± 4.7	< 0.001	41.1 ± 5.9	43.6 ± 6.0	0.001
Last follow‐up BMI, kg/m^2^	31.6 ± 6.4	37.3 ± 6.3	28.8 ± 4.3	< 0.001	33.7 ± 5.9	30.3 ± 6.2	< 0.001
Metabolic comorbidities							
Hypertension, *n* (%)	120 (34.1)	50 (45.9)	70 (28.9)	0.004	27 (29.7)	69 (36.5)	0.276
Diabetes mellitus, *n* (%)	46 (13.1)	20 (18.3)	26 (10.7)	0.048	7 (7.6)	29 (15.3)	0.076
Dyslipidemia, *n* (%)	167 (47.6)	50 (45.9)	117 (48.3)	0.674	38 (41.7)	97 (51.3)	0.145
Insulin resistance, *n* (%)	299 (85.2)	89 (81.7)	210 (86.8)	0.422	69 (75.8)	170 (89.9)	0.017
Bariatric procedure, *n* (%)				< 0.001			< 0.001
Gastric banding	158 (45.0)	80 (73.4)	78 (32.2)		61 (67.0)	69 (36.6)	
Gastric bypass	143 (40.7)	22 (20.2)	121 (50.0)		22 (24.2)	87 (46.0)	
Sleeve gastrectomy	40 (11.4)	3 (2.8)	37 (15.3)		6 (6.6)	28 (14.8)	
Gastric plication/other	10 (2.9)	4 (3.6)	6 (2.5)		2 (2.2)	5 (2.6)	
Previous intragastric balloon, *n* (%)	95 (27.1)	46 (42.2)	49 (20.2)	< 0.001	26 (28.6)	52 (27.5)	0.726

*Note:* Data are reported as mean ± SD or median (IQR) for continuous variables, according to their distribution, and as *n* (%) for categorical variables.

Abbreviations: BMI, body mass index; IWL, insufficient weight loss; WR, weight regain.

^a^ WR data were available for 280 patients.

^b^ Significance for *p* value < 0.05.

### 3.2 Insufficient Weight Loss

IWL had a prevalence of 31.1% (Table 1). Patients with IWL had higher preoperative BMI than those without IWL (45.8 ± 7.4 vs. 41.6 ± 4.7 kg/m^2^, *p* < 0.001) and were more frequently affected by class III obesity (74.3% vs. 58.3%; *p* < 0.001), whereas class II obesity was less common (23.9% vs. 37.6%; *p* < 0.001). Patients with IWL also had a higher prevalence of hypertension (45.9% vs. 28.9%, *p* = 0.004), T2DM (18.3% vs. 10.7%, *p* = 0.048), and psychiatric comorbidity (16.5% vs. 7.4%, *p* = 0.017). Other baseline characteristics did not differ significantly.

Bariatric procedure differed significantly by IWL status. Gastric banding was more frequent among patients with IWL (73.4% vs. 32.2%, *p* < 0.001), whereas gastric bypass (20.2% vs. 50.0%, *p* < 0.001) and sleeve gastrectomy (2.8% vs. 15.3%, *p* < 0.001) were less frequent. Previous intragastric balloon use was also more common among patients experiencing IWL (42.2% vs. 20.2%, *p* < 0.001). Laboratory parameters did not differ significantly between groups (Table S1).

As for histological features (Table 2), MASLD, MASH, and fibrotic MASH were not associated with IWL. Conversely, fibrosis stage differed between groups (*p* = 0.012), with significant fibrosis stage ≥ 2 being more frequent in patients with IWL compared with those without IWL (13.8% vs. 4.5%, *p* = 0.002).

**TABLE 2:** Liver histology according to insufficient weight loss and weight regain status.

Variables	Whole cohort (*n* = 351)	IWL (*n* = 109)	No IWL (*n* = 242)	*p*	WR (*n* = 91)^a^	No WR (*n* = 189)^a^	*p*
Steatosis grade, *n* (%)				0.475			0.469
Grade 0	51 (14.5)	15 (13.7)	36 (14.9)		15 (16.4)	26 (13.8)	
Grade 1	124 (35.3)	36 (33.0)	88 (36.4)		35 (38.5)	62 (32.8)	
Grade 2	92 (26.2)	26 (23.9)	66 (27.3)		23 (25.3)	48 (25.4)	
Grade 3	84 (24.0)	32 (29.4)	52 (21.4)		18 (19.8)	53 (28.0)	
Lobular inflammation grade, *n* (%)				0.820			0.052
Grade 0	127 (36.2)	39 (35.8)	88 (36.4)		37 (40.7)	60 (31.7)	
Grade 1	178 (50.7)	56 (51.4)	122 (50.4)		49 (53.8)	97 (51.3)	
Grade 2	44 (12.5)	14 (12.8)	30 (12.4)		5 (5.5)	30 (15.9)	
Grade 3	2 (0.6)	0 (0.0)	2 (0.8)		0 (0.0)	2 (1.1)	
Ballooning grade, *n* (%)				0.071			0.097
Grade 0	267 (76.0)	91 (83.5)	176 (72.7)		74 (81.3)	132 (69.8)	
Grade 1	82 (23.4)	18 (16.5)	64 (26.5)		17 (18.7)	55 (29.1)	
Grade 2	2 (0.6)	0 (0.0)	2 (0.8)		0 (0.0)	2 (1.1)	
Fibrosis stage, *n* (%)				0.012			0.011
F0	211 (60.0)	58 (53.2)	153 (63.3)		66 (72.5)	102 (53.9)	
F1	114 (32.5)	36 (33.0)	78 (32.2)		24 (26.4)	66 (34.9)	
F2	20 (5.7)	10 (9.2)	10 (4.1)		1 (1.1)	15 (8.0)	
F3	3 (0.9)	3 (2.8)	0 (0.0)		0 (0.0)	3 (1.6)	
F4	3 (0.9)	2 (1.8)	1 (0.4)		0 (0.0)	3 (1.6)	
NAS	3.0 (1.0–4.0)	3.0 (1.0–4.0)	2.0 (1.0–4.0)	0.872	2.0 (1.0–4.0)	3.0 (1.5–4.0)	—
MASLD, *n* (%)	300 (85.5)	94 (86.2)	206 (85.1)	0.761	76 (83.5)	163 (86.2)	0.545
MASH, *n* (%)	43 (12.2)	9 (8.3)	34 (14.0)	0.123	9 (9.9)	31 (16.4)	0.145
Fibrotic MASH, *n* (%)	32 (9.1)	9 (8.3)	23 (9.5)	0.699	6 (6.6)	23 (12.1)	0.152
Fibrosis, *F* ≥ 1, *n* (%)	139 (39.6)	51 (46.8)	88 (36.4)	0.069	25 (27.5)	88 (46.6)	0.003
Significant fibrosis, *F* ≥ 2, *n* (%)	26 (7.4)	15 (13.8)	11 (4.5)	0.002	1 (1.1)	21 (11.1)	0.004

*Note:* Data are reported as mean ± SD or median (IQR) for continuous variables, according to their distribution, and as *n* (%) for categorical variables.

Abbreviations: IQR, interquartile range; IWL, insufficient weight loss; MASH, metabolic dysfunction–associated steatohepatitis; MASLD, metabolic dysfunction–associated steatotic liver disease; NAS, NAFLD activity score; WR, weight regain.

^a^ WR data were available for 280 patients.

PNPLA3, TM6SF2, GCKR, and HSD17B13 variants were not associated with IWL (Table 3). IRS1 rs2972146 distribution differed by IWL status (*p* for trend = 0.050), with variant carriage less frequent in patients with IWL (78.8% vs. 88.8%, *p* = 0.021).

**TABLE 3:** Distribution of MASLD‐related genetic variants according to insufficient weight loss and weight regain status.

Variables	Whole cohort (*n* = 351)	IWL (*n* = 109)	No IWL (*n* = 242)	*p*	WR (*n* = 91)^a^	No WR (*n* = 189)^a^	*p*
*Genetic variants*							
PNPLA3 rs738409 G allele, *n* (%)				0.539			0.510
Non‐carrier	167 (47.6)	55 (50.4)	112 (46.3)		42 (46.1)	89 (47.1)	
Heterozygous carrier	146 (41.6)	44 (40.4)	102 (42.1)		38 (41.9)	78 (41.3)	
Homozygous carrier	38 (10.8)	10 (9.2)	28 (11.6)		11 (12.0)	22 (11.6)	
TM6SF2 rs58542926 T allele, *n* (%)				0.081			0.695
Non‐carrier	324 (92.3)	97 (89)	227 (94.0)		84 (92.3)	177 (93.7)	
Heterozygous carrier	25 (7.1)	11 (10.1)	14 (5.6)		7 (7.7)	12 (6.3)	
Homozygous carrier	2 (0.6)	1 (0.9)	1 (0.4)		0 (0.0)	0 (0.0)	
GCKR rs1260326, *n* (%)				0.399			0.393
Genotype 1	85 (24.2)	23 (21.1)	62 (25.6)		21 (23.1)	46 (24.3)	
Genotype 2	171 (48.8)	54 (49.5)	117 (48.3)		41 (45.0)	98 (51.9)	
Genotype 3	95 (27.0)	32 (29.4)	63 (26.1)		29 (31.9)	45 (23.8)	
HSD17B13 rs72613567 TA allele, *n* (%)				0.609			0.746
Non‐carrier	225 (64.1)	69 (63.3)	156 (64.5)		61 (67.0)	120 (63.5)	
Heterozygous carrier	108 (30.8)	32 (29.4)	76 (31.4)		26 (28.6)	62 (32.8)	
Homozygous carrier	18 (5.1)	8 (7.3)	10 (4.1)		4 (4.4)	7 (3.7)	
IRS1 rs2972146, *n* (%)				0.050			0.859
Non‐carrier	50 (14.2)	23 (21.1)	27 (11.2)		15 (16.5)	26 (13.8)	
Heterozygous carrier	168 (47.9)	46 (42.2)	122 (50.4)		41 (45.1)	90 (47.5)	
Homozygous carrier	133 (37.9)	40 (36.7)	93 (38.4)		35 (38.4)	73 (38.7)	

*Note:* Data are reported as mean ± SD or median (IQR) for continuous variables, according to their distribution, and as *n* (%) for categorical variables.

Abbreviations: GCKR, glucokinase regulator; HSD17B13, hydroxysteroid 17‐beta dehydrogenase 13; IRS1, insulin receptor substrate 1; IWL, insufficient weight loss; MASLD, metabolic dysfunction–associated steatotic liver disease; PNPLA3, patatin‐like phospholipase domain‐containing protein 3; TM6SF2, transmembrane 6 superfamily member 2; WR, weight regain.

^a^ WR data were available for 280 patients.

To disentangle baseline host‐related factors from procedure‐ and lifestyle‐related determinants of IWL, two multivariable logistic regression models were fitted (Figure 1). Model A included preoperative host‐related variables associated with IWL or considered clinically relevant and was adjusted for age, sex, preoperative BMI, psychiatric comorbidity, hypertension, fibrosis stage ≥ 2, and IRS1 rs2972146 variant carrier status (Figure 1, Model A). In this model, hypertension (OR 1.97, 95% CI: 1.06–3.63; *p* = 0.031) and fibrosis stage ≥ 2 (OR 2.93, 95% CI: 1.07–8.01; *p* = 0.036) were independently associated with higher odds of IWL, whereas IRS1 rs2972146 variant was protective against IWL (OR 0.30, 95% CI: 0.14–0.62; *p* = 0.001).

**FIGURE 1:**
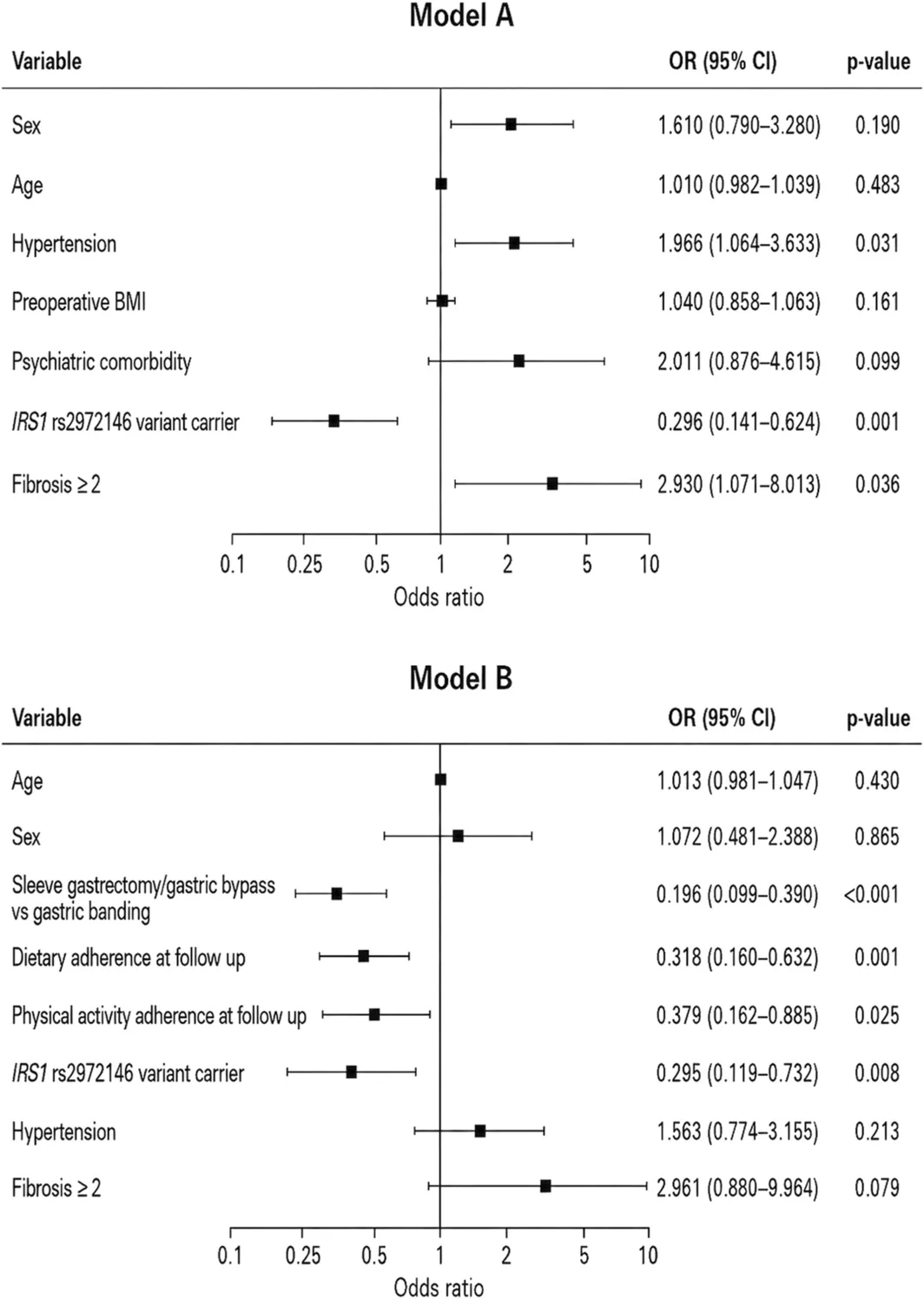
Multivariable logistic regression models assessing factors associated with insufficient weight loss. Forest plots show adjusted odds ratios (ORs) and 95% confidence intervals (CIs) for factors associated with insufficient weight loss. Model A evaluated baseline host‐related factors only, including age, sex, preoperative BMI, psychiatric comorbidity, hypertension, significant fibrosis, and IRS1 rs2972146 variant carrier status. Model B extended Model A by additionally including procedure‐ and postoperative lifestyle‐related factors, namely bariatric procedure type, dietary adherence at follow‐up, and physical activity adherence at follow‐up. BMI, body mass index; CI, confidence interval; insufficient weight loss; IRS‐1, insulin receptor substrate 1; OR, odds ratio.

Model B additionally included procedure‐ and postoperative lifestyle‐related factors, including bariatric procedure type, dietary adherence, and physical activity adherence at follow‐up, while adjusting for age, sex, and the host‐related variables independently associated with IWL in Model A, namely hypertension, fibrosis stage ≥ 2, and IRS1 rs2972146 variant carrier status (Figure 1, Model B). In this model, sleeve gastrectomy or gastric bypass (OR 0.20, 95% CI: 0.10–0.39; *p* < 0.001), dietary adherence at follow‐up (OR 0.32, 95% CI: 0.16–0.63; *p* = 0.001), physical activity adherence at follow‐up (OR 0.38, 95% CI: 0.16–0.89; *p* = 0.025), and IRS1 rs2972146 variant carrier status (OR 0.30, 95% CI: 0.12–0.73; *p* = 0.008) were independently associated with lower odds of IWL.

In the sensitivity analysis using the weight‐based component of the 2024 IFSO definition, 83 of 351 patients (23.6%) had a suboptimal initial response. Two adjusted multivariable logistic regression models were fitted (Figure S1). In Model A, significant fibrosis was independently associated with higher odds of suboptimal initial response (OR 4.981, 95% CI: 1.899–13.069; *p* = 0.030), whereas IRS1 rs2972146 variant carrier status was associated with lower odds (OR 0.398, 95% CI: 0.185–0.859; *p* = 0.019). In Model B, dietary adherence at follow‐up (OR 0.134, 95% CI: 0.062–0.290; *p* < 0.001) and IRS1 rs2972146 variant carrier status (OR 0.375, 95% CI: 0.143–0.981; *p* = 0.046) were independently associated with lower odds of suboptimal initial response.

### 3.3 Weight Regain

Among the 351 patients assessed for IWL, 280 had available data on postoperative weight nadir and subsequent follow‐up weight and were therefore included in the WR analysis (Table 1). WR occurred in 91 patients (32.5%) after a median follow‐up of 43 months (IQR 24–98). Patients with WR had lower preoperative BMI than those without WR (41.1 ± 5.9 vs. 43.6 ± 6.0 kg/m^2^, *p* = 0.001). Preoperative BMI class differed by WR status, with patients with WR more frequently in the BMI 35–40 kg/m^2^ class (46.2% vs. 27.0%) and less frequently in the BMI ≥ 40 kg/m^2^ class (46.2% vs. 70.9%; overall *p* < 0.001). WR was also associated with psychiatric comorbidity (17.6% vs. 7.9%, *p* = 0.012), whereas insulin resistance was less frequent among patients with WR (75.8% vs. 89.9%, *p* = 0.017). Dietary adherence at last follow‐up was also less frequent among patients with WR than among those without WR (33.0% vs. 65.6%, *p* < 0.001). No significant differences were observed for sex, smoking, physical activity, hypertension, T2DM, dyslipidemia, or baseline pharmacological treatments.

Bariatric procedure differed significantly by WR status. Gastric banding was more frequent among patients with WR (67.0% vs. 36.6%), whereas gastric bypass (24.2% vs. 46.0%) and sleeve gastrectomy (6.6% vs. 14.8%) were less frequent (overall *p* < 0.001). Conversely, the rate of previous intragastric balloon procedures was similar between groups (28.6% vs. 27.5%, *p* = 0.726).

Patients with WR had lower glucose, HOMA‐IR, ALT, AST, and ferritin levels; other laboratory parameters were similar (Table S1).

As for histological features (Table 2), MASLD, MASH, and fibrotic MASH were not associated with WR. However, overall fibrosis stage differed between groups (*p* = 0.011), with fibrosis stage ≥ 1 and fibrosis stage ≥ 2 less frequent among patients with WR than among those without WR (27.5% vs. 46.6%, *p* = 0.003; and 1.1% vs. 11.1%, *p* = 0.004, respectively). No significant differences in PNPLA3 rs738409, TM6SF2 rs58542926, GCKR rs1260326, HSD17B13 rs72613567, or IRS1 rs2972146 distribution were observed according to WR status (Table 3).

To disentangle baseline host‐related factors from procedure‐ and lifestyle‐related determinants of WR, two multivariable logistic regression models were fitted (Figure 2). Model A included baseline host‐related variables associated with WR or considered clinically relevant, and was adjusted for age, sex, insulin resistance, preoperative BMI, and psychiatric comorbidity (Figure 2, Model A). In this model, lower preoperative BMI was independently associated with higher odds of WR (OR 0.89, 95% CI: 0.81–0.97; *p* = 0.007), whereas psychiatric comorbidity was independently associated with higher odds of WR (OR 3.31, 95% CI: 1.20–9.18; *p* = 0.021).

**FIGURE 2:**
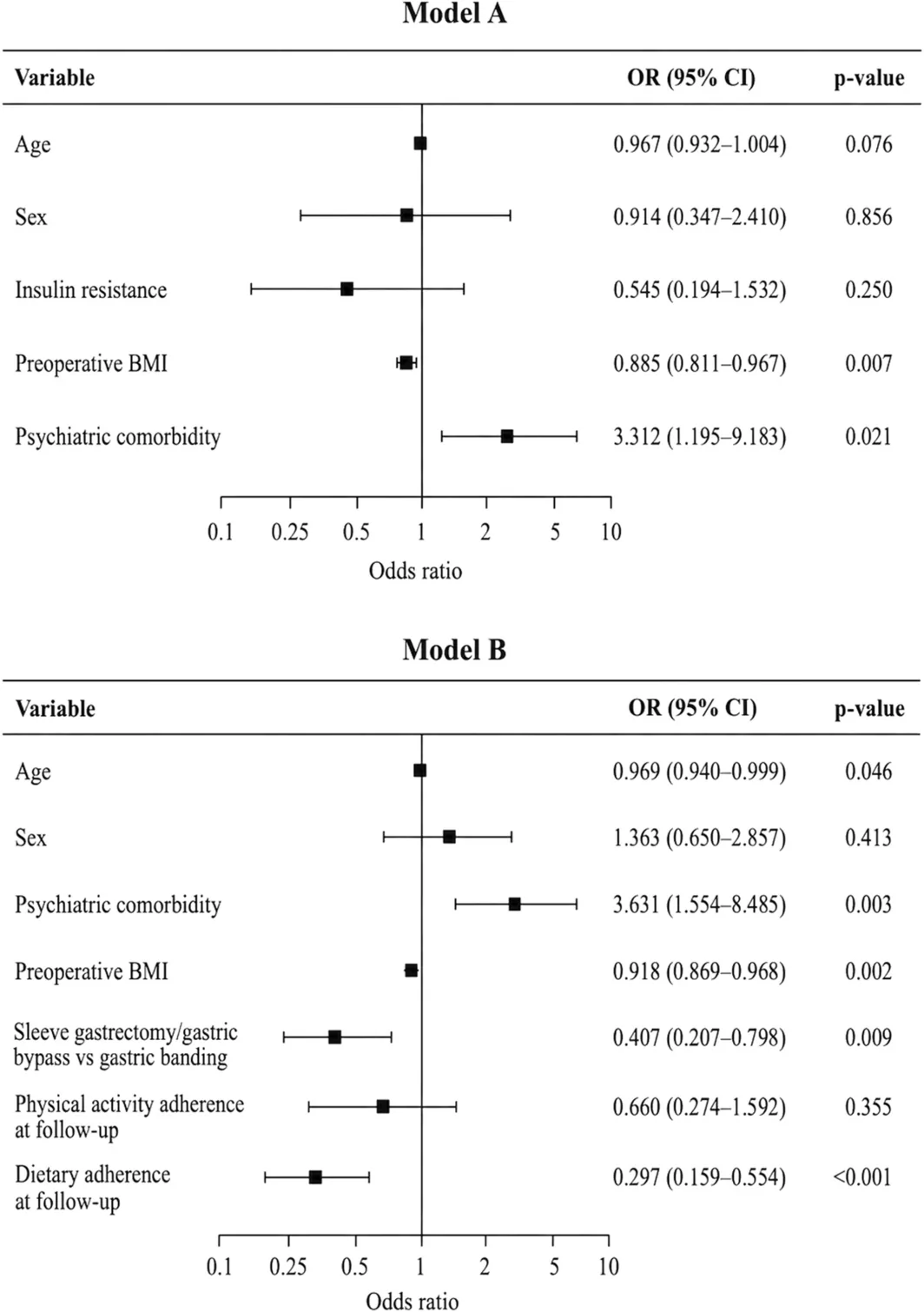
Multivariable logistic regression models assessing factors associated with weight regain. Forest plots show adjusted odds ratios (ORs) and 95% confidence intervals (CIs) for factors associated with weight regain. Model A evaluated baseline host‐related factors only, including age, sex, insulin resistance, preoperative BMI, and psychiatric comorbidity. Model B extended Model A by additionally including procedure‐ and postoperative lifestyle‐related factors, namely bariatric procedure type, dietary adherence at follow‐up, and physical activity adherence at follow‐up. BMI, body mass index; CI, confidence interval; OR, odds ratio; WR, weight regain.

Model B additionally included procedure‐ and postoperative lifestyle‐related factors, including bariatric procedure type, dietary adherence, and physical activity adherence at follow‐up, while adjusting for age, sex, and the host‐related variables independently associated with WR in Model A, namely preoperative BMI and psychiatric comorbidity (Figure 2, Model B). In this model, psychiatric comorbidity remained independently associated with higher odds of WR (OR 3.63, 95% CI: 1.55–8.49; *p* = 0.003). Conversely, higher preoperative BMI was associated with lower odds of WR (OR 0.92, 95% CI: 0.87–0.97; *p* = 0.002), as were sleeve gastrectomy or gastric bypass (OR 0.41, 95% CI: 0.21–0.80; *p* = 0.009) and dietary adherence at follow‐up (OR 0.30, 95% CI: 0.16–0.55; *p* < 0.001).

In the sensitivity analysis using the weight‐based component of the 2024 IFSO definition, 74 of 280 patients (26.4%) met the criteria for late postoperative clinical deterioration. Two adjusted multivariable logistic regression models were fitted (Figure S2). In Model A, higher preoperative BMI was independently associated with lower odds of suboptimal initial response (OR 0.902, 95% CI: 0.828–0.982), whereas psychiatric comorbidity was associated with higher odds (OR 3.082, 95% CI: 1.049–9.052). In Model B, higher preoperative BMI remained independently associated with lower odds of suboptimal initial response (OR 0.899, 95% CI: 0.849–0.952), as did physical activity adherence (OR 0.304, 95% CI: 0.125–0.737) and dietary adherence (OR 0.209, 95% CI: 0.108–0.405; *p* < 0.001).

### 3.4 Temporal Analysis

Temporal analyses included 171 patients operated on in 2008–2016 and 180 in 2017–2023. Surgical practice changed substantially between periods (*p* < 0.001): gastric banding declined from 70.2% to 21.1%, while gastric bypass increased from 25.1% to 55.6% and sleeve gastrectomy from 0.6% to 21.7%. IWL was less frequent in the later period (17.2% vs. 45.6%, *p* < 0.001), whereas WR prevalence remained similar (29.4% vs. 35.8%, *p* = 0.31). The inverse association between IRS1 rs2972146 carrier status and IWL was directionally consistent across periods but reached statistical significance only in 2008–2016 (OR 0.29, 95% CI: 0.09–0.94; 2017–2023: OR 0.34, 95% CI: 0.07–1.70). Significant fibrosis was less prevalent in the later period (4.4% vs. 10.5%); however, the small number of cases precluded reliable period‐specific estimates of its association with IWL.

## 4 Discussion

In this cohort of patients undergoing MBS with systematic intraoperative liver biopsy, weight loss failure affected nearly one third of patients, underscoring that suboptimal postoperative response remains common despite the overall efficacy of surgery. Suboptimal weight loss after MBS appears multifactorial, with cardiometabolic and liver‐related burden, host genetics, procedure type, psychological factors and lifestyle adherence contributing differently to early IWL and later WR. Importantly, significant liver fibrosis and IRS1 rs2972146 wild‐type variant resulted independent risk factors for IWL, highlighting a new role for liver disease severity as a marker of impaired early weight‐loss response after MBS, while identifying IRS1 rs2972146 as a novel host genetic signal potentially linking insulin signalling, adipose tissue biology, and postoperative metabolic adaptation.

The prevalence of weight loss failure was consistent with previous reports (15%–35%) [10, 30], although comparisons are limited by heterogeneous definitions, procedures, and follow‐up [10, 14, 31]. The finding that nearly one third of patients experienced suboptimal postoperative weight outcomes highlights the clinical relevance of weight loss failure after MBS. To better characterise its determinants, we distinguished baseline host‐related metabolic and biological factors from modifiable procedural and lifestyle factors.

Among host‐related factors, the most novel finding was the independent association between significant liver fibrosis and IWL. This finding was supported by the sensitivity analysis showing higher odds of IFSO‐defined suboptimal initial response among patients with significant fibrosis. Although several preoperative comorbidities, including T2DM, hypertension, obstructive sleep apnea, dyslipidemia, and psychiatric comorbidity, have been linked to poorer weight loss after MBS [10, 14, 17], the role of liver disease remains less well defined. Prior biopsy‐based studies have suggested that baseline MASLD or MASH may be associated with lower postoperative weight lossloss [32, 33], whereas others found no association between fibrosis stage, or even Child A cirrhosis, and postoperative weight reduction [34, 35]. In our cohort, significant liver fibrosis, but not MASLD or MASH, was independently associated with IWL, suggesting that fibrosis may identify patients less likely to achieve expected early weight loss targets. Biologically, liver fibrosis in patients living with obesity may act as an integrated marker of long‐standing cardiometabolic dysfunction, reflecting cumulative insulin resistance, chronic inflammation, and adipose tissue dysfunction [36], which may reduce metabolic adaptability after surgery. However, the number of patients with significant fibrosis was relatively small; therefore, this novel association warrants further investigation in larger prospective cohorts. Hypertension was also independently associated with IWL, consistent with reports linking cardiometabolic burden to poorer postoperative weight loss [10, 14, 31, 37]. However, rather than a direct causal factor, hypertension may reflect advanced metabolic disease characterized by insulin resistance, vascular inflammation, neurohormonal activation, and reduced cardiometabolic flexibility [38].

Beyond histological liver disease severity, we evaluated selected genetic variants involved in MASLD susceptibility and progression, liver fibrosis and insulin resistance. PNPLA3, TM6SF2, GCKR, and HSD17B13 mutations were not associated with IWL or WR, whereas IRS1 rs2972146 was the only variant independently associated with weight loss failure after MBS. Importantly, the association between IRS1 rs2972146 carrier status and lower IWL risk persisted for IFSO‐defined suboptimal initial response in both sensitivity models, supporting its robustness across definitions. This finding is novel and biologically plausible, although exploratory. IRS1 encodes insulin receptor substrate 1, a key adaptor of insulin signalling, and variants near IRS1 have been linked to adiposity, insulin resistance, dyslipidemia, T2DM, and coronary artery disease, suggesting that this locus may modulate the interface between fat distribution, metabolic health, and response to weight‐loss interventions [29, 39]. Prior genetic and functional evidence further supports the relevance of IRS1 rs2972146. In a GWAS meta‐analysis of more than 100 000 individuals, Teslovich et al. identified rs2972146 as a genome‐wide significant lipid‐associated variant related to HDL cholesterol and triglycerides, and linked it to IRS1 expression in visceral adipose tissue, suggesting a role in adipose biology and cardiometabolic risk [40]. Moreover, rs2972146 is in high linkage disequilibrium with rs2943650, another variant located near IRS1, associated with body fat percentage and metabolic traits [41]. Evidence from different populations suggests that IRS1 rs2943650 variants may influence the capacity of adipose tissue to safely store excess lipids. Impaired adipose expandability may promote ectopic fat deposition in non‐adipose organs, contributing to insulin resistance, dyslipidemia, type 2 diabetes, and cardiovascular disease despite lower overall adiposity [41, 42].

Together with intervention studies [43, 44], these findings support a role for IRS1 in adipose distribution, insulin signalling, and metabolic flexibility.

To our knowledge, this is the first study linking IRS1 rs2972146 to early weight loss response after MBS, suggesting that genetically determined differences in insulin signalling and adipose tissue function may contribute to postoperative weight‐loss heterogeneity. However, replication in larger and ethnically diverse bariatric cohorts is warranted.

Among host‐related factors associated with WR, psychiatric comorbidity emerged as the most clinically relevant determinant. This finding was supported by the sensitivity analysis, in which psychiatric comorbidity was associated with higher odds of IFSO‐defined late postoperative clinical deterioration in the host‐related model. Although its relationship with postoperative weight outcomes remains heterogeneous across studies [45, 46, 47], psychiatric comorbidity may promote WR indirectly through emotional eating, loss‐of‐control eating, impaired emotion regulation, reduced adherence to dietary and behavioural recommendations, psychotropic medication‐related weight gain, and lower engagement with postoperative care [10, 14]. Thus, rather than representing a simple baseline characteristic, psychiatric comorbidity may identify patients who require closer long‐term multidisciplinary follow‐up, including structured psychological, nutritional, and pharmacological support to prevent weight recurrence.

Lower preoperative BMI was also independently associated with WR in our cohort. This contrasts with previous reports linking higher baseline BMI to poorer postoperative weight rajectories [48, 49, 50], and with our own univariable analysis, in which higher BMI was associated with IWL. However, this finding should be interpreted cautiously, as WR estimates are highly definition‐dependent. When WR is defined as a relative increase from postoperative nadir, patients with lower absolute body weight may reach the prespecified percentage threshold with a smaller absolute gain. Thus, lower preoperative BMI may reflect the metric used to define WR rather than a true biological predisposition to regain weight. This reinforces the need for standardized, clinically meaningful WR definitions that integrate relative and absolute weight regain, weight trajectory, and recurrence of obesity‐related comorbidities.

Among procedure‐ and lifestyle‐related factors, Gastric banding and poor dietary adherence were independently associated with both IWL and WR, while poor physical activity adherence was additionally associated with IWL. The dietary findings were confirmed in the sensitivity analyses, in which adherence was associated with lower odds of both IFSO‐defined outcomes. Physical activity adherence was associated with lower odds of late postoperative clinical deterioration and showed a similar, although non‐significant, association with suboptimal initial response. The association with gastric banding is established [51, 52, 53, 54], and likely reflects both the historical case mix and limitations of a purely restrictive procedure. Postoperative behavioural factors remained associated with outcomes after adjustment, reinforcing that MBS requires structured long‐term care. Dietary non‐adherence, maladaptive eating, physical inactivity, and poor follow‐up contribute to weight loss failure [10, 55, 56, 57, 58]. Sustained nutritional counselling, physical activity support, and multidisciplinary follow‐up are therefore essential to consolidate the metabolic effects of surgery and prevent weight recurrence [59].

Temporal analyses reflected the evolution of bariatric practice, with a marked shift from gastric banding toward gastric bypass and sleeve gastrectomy. The lower prevalence of IWL in the later period likely reflects the greater effectiveness of contemporary procedures, together with changes in surgical indications, patient selection, nutritional follow‐up, and anti‐obesity pharmacotherapy.

Strengths of the study include the real‐world cohort with systematic liver biopsy, evaluation of genetic variants related to MASLD and insulin resistance, and separate analysis of IWL and WR, integrating clinical, histological, genetic, procedural, psychiatric, and lifestyle factors.

Limitations include the retrospective, single‐center design, with potential residual confounding information bias related to the use of clinical records and selection bias. The long recruitment period (2008–2023) may have introduced heterogeneity in surgical practices, patient selection, follow‐up, and pharmacotherapy, with residual era‐related confounding despite temporal analyses. The high proportion of gastric banding, now rarely performed and associated with poorer weight‐loss outcomes, limits external validity and may introduce residual confounding despite multivariable analysis adjustment for procedure type. WR data were available for fewer patients potentially introducing attrition bias and reducing statistical precision; lifestyle adherence was assessed from clinical records, and detailed information on eating behaviours, body composition, energy expenditure, and exercise type was not systematically available. Complete IFSO definitions could not be applied because longitudinal complication data were unavailable; however, sensitivity analyses were conducted using their weight‐based components. Finally, the limited number of patients with significant fibrosis reduced statistical precision, while the genetic analyses were exploratory. Accordingly, the associations of significant fibrosis and IRS1 rs2972146 with IWL should be considered hypothesis‐generating and require external validation.

In conclusion, weight loss failure affected nearly one third of patients undergoing MBS. Significant liver fibrosis and hypertension identified higher IWL risk, whereas psychiatric comorbidity was the main host‐related determinant of WR. Gastric banding and poor dietary adherence were associated with both outcomes, and poor physical activity adherence with IWL. IRS1 rs2972146 carrier status was associated with lower IWL odds, suggesting a potential role for insulin signalling and adipose biology. These findings support a precision‐based approach to MBS, integrating liver disease severity, genetic susceptibility, procedure selection, and structured multidisciplinary follow‐up to optimize long‐term outcomes.

## Author Contributions

F.C., A.L.F., P.D., and R.L. conceived and designed the study. M.M., M.L., E.P., and P.D. performed the genetic analyses and contributed to data acquisition and curation. G.O., G.P., G.M., M.M., E.C., V.S., E.C., F.A., A.C., and F.C. contributed to clinical data acquisition and curation. S.B., E.G., and L.B. contributed to the surgical aspects of the study, including surgical data acquisition and interpretation. F.C. and R.L. curated the clinical dataset, performed the statistical analyses, interpreted the data, and drafted the manuscript. A.L.F., P.D., L.B., and R.L. supervised the study and critically revised the manuscript for important intellectual content. All authors reviewed and approved the final version of the manuscript.

## Funding

The authors have nothing to report.

## Ethics Statement

The study was approved by the Ethics Committee of Fondazione IRCCS Ca′ Granda Ospedale Maggiore Policlinico, Milan, Italy (Study ce 401). The research was conducted in accordance with the principles of the Declaration of Helsinki.

## Consent

All participants provided informed consent before enrollment.

## Conflicts of Interest

A.L.F. had a role as PI in pharmacological trials from Boeringher Ingelhein, Novo Nordisk, MSD, AstraZenecam, GSK; R.L. had a role as consultant for Boeringher Ingelhein and Business Integration Partner S.P.A. and has received research grants from Gilead. The other authors declare no conflicts of interest.

## Supporting information


**Table S1:** Baseline laboratory parameters in the overall cohort and according to insufficient weight loss and weight regain status.
**Figure S1:** Multivariable logistic regression models assessing factors associated with suboptimal initial response.
**Figure S2:** Multivariable logistic regression models assessing factors associated with late post‐operative clinical deterioration.

## Data Availability

The datasets generated and analysed during the current study are not publicly available due to privacy and ethical restrictions, but are available from the corresponding author on reasonable request and subject to approval by the relevant institutional and ethics authorities.
