# Supplementary material for: Liver Fibrosis and IRS1 rs2972146 Are Associated With Weight Loss Response After Metabolic Bariatric Surgery
Source: Diabetes Obes Metab. 2026 Sep 17;28(12):11532–44. doi: 10.1111/dom.71345 (PMC13731044; doi:10.1111/dom.71345)
Supplement: Supplementary file 1 [file DOM-28-11532-s001.docx]

**Supplementary Table 1:** Baseline laboratory parameters in the overall cohort and according to insufficient weight loss and weight regain status.

| **Variables** | **Whole cohort (n=351)** | **IWL (n=109)** | **No IWL (n=242)** | **p-value** | **WR (n=91)#** | **No WR (n=189)#** | **p-value** |
| --- | --- | --- | --- | --- | --- | --- | --- |
| Total cholesterol, mg/dL | 196.6 ± 39.2 | 198.5 ± 42.4 | 195.9 ± 37.9 | 0.619 | 201.1 ± 42.9 | 196.2 ± 39.9 | 0.408 |
| Triglycerides, mg/dL | 112.0 (80.8–161.3) | 115 (77–165) | 109.0 (81.3–158.3) | 0.569 | 101.0 (68.5–148.5) | 117.0 (87.0–165.3) | 0.054 |
| HDL cholesterol, mg/dL | 51.1 ± 14.1 | 50.7 ± 12.5 | 51.2 ± 14.7 | 0.808 | 52.5 ± 18.2 | 51.0 ± 13.2 | 0.501 |
| LDL cholesterol, mg/dL | 127.0 ± 36.7 | 122.9 ± 38.1 | 128.6 ± 36.0 | 0.246 | 132.0 ± 37.0 | 126.2 ± 38.0 | 0.292 |
| Glucose, mg/dL | 100.6 ± 21.7 | 103.1 ± 24.5 | 99.5 ± 20.3 | 0.148 | 96.7 ± 14.9 | 102.4 ± 23.1 | 0.033 |
| HbA1c, mmol/mol | 40.1 ± 9.9 | 41.5 ± 8.7 | 39.4 ± 10.4 | 0.147 | 38.3 ± 6.6 | 40.5 ± 10.3 | 0.158 |
| HOMA-IR index | 5.1 (3.3–7.7) | 5.03 (3.1–8.7) | 5.1 (3.3–7.3) | 0.695 | 4.4 (2.6–6.8) | 5.4 (3.7–8.4) | 0.046 |
| ALT, U/L | 22.0 (17.0–34.0) | 21 (17–36) | 23.0 (17.0–34.0) | 0.996 | 20.0 (15.0–32.0) | 25.0 (17.0–36.0) | 0.003 |
| AST, U/L | 20.0 (16.0–26.0) | 19 (16–25) | 20.5 (16.0–26.0) | 0.380 | 18.0 (15.0–22.3) | 21.0 (17.0–27.0) | <0.001 |
| GGT, U/L | 23.0 (15.0–38.3) | 24 (17–35) | 23.0 (14.0–40.0) | 0.439 | 22.0 (15.0–40.0) | 23.0 (14.0–36.9) | 0.628 |
| Ferritin, ng/mL | 87.0 (41.0–168.0) | 89 (36–168) | 86.0 (44.0–169.5) | 0.703 | 60.5 (33.3–152.0) | 94.5 (53.8–186.0) | 0.026 |

Data are reported as mean ± SD or median (IQR), according to their distribution. #Data on weight regain were available for 280 patients.

**Abbreviations:** ALT, alanine aminotransferase; AST, aspartate aminotransferase; GGT, gamma-glutamyl transferase; HbA1c, glycated hemoglobin; HDL, high-density lipoprotein; HOMA-IR, homeostatic model assessment of insulin resistance; IQR, interquartile range; IWL, insufficient weight loss; LDL, low-density lipoprotein; SD, standard deviation; TSH, thyroid-stimulating hormone; WR, weight regain.

Supplementary Figure 1: Multivariable logistic regression models assessing factors associated with suboptimal initial response.

**Note:** Forest plots show adjusted odds ratios (ORs) and 95% confidence intervals (CIs) for factors associated with insufficient weight loss. **Model A** evaluated baseline host-related factors only, including age, sex, preoperative BMI, psychiatric comorbidity, hypertension, significant fibrosis, and IRS1 rs2972146 variant carrier status. **Model B** extended Model A by additionally including procedure- and postoperative lifestyle-related factors, namely bariatric procedure type, dietary adherence at follow-up, and physical activity adherence at follow-up. **Abbreviations:** BMI, body mass index; CI, confidence interval; insufficient weight loss; IRS-1, insulin receptor substrate 1 ; OR, odds ratio.

Supplementary Figure 2: Multivariable logistic regression models assessing factors associated with late post-operative clinical deterioration.

**Note:** Forest plots show adjusted odds ratios (ORs) and 95% confidence intervals (CIs) for factors associated with weight regain. Model A evaluated baseline host-related factors only, including age, sex, insulin resistance, preoperative BMI, and psychiatric comorbidity. Model B extended Model A by additionally including procedure- and postoperative lifestyle-related factors, namely bariatric procedure type, dietary adherence at follow-up, and physical activity adherence at follow-up.

**Abbreviations:** BMI, body mass index; CI, confidence interval; OR, odds ratio; WR, weight regain.
